# The Development of Flexible Problem Solving: An Integrative Approach

**DOI:** 10.3390/jintelligence11060119

**Published:** 2023-06-14

**Authors:** Katarzyna Bobrowicz, Jean-Pierre Thibaut

**Affiliations:** 1 Department of Behavioural and Cognitive Sciences, University of Luxembourg, 4366 Esch-sur-Alzette, Luxembourg; 2 LEAD—Centre National de la Recherche Scientifique UMR-5022, University of Burgundy, 21000 Dijon, France; jean-pierre.thibaut@u-bourgogne.fr

**Keywords:** memory generalization, analogical transfer, representational flexibility, executive functions, cognitive development

## Abstract

Flexible problem solving, the ability to deal with currently goal-irrelevant information that may have been goal-relevant in previous, similar situations, plays a prominent role in cognitive development and has been repeatedly investigated in developmental research. However, this research, spanning from infancy to the school years, lacks a unifying framework, obscuring the developmental timing of flexible problem solving. Therefore, in this review paper, previous findings are gathered, organized, and integrated under a common framework to unveil how and when flexible problem solving develops. It is showed that the development of flexible problem solving coincides with increases in executive functions, that is, inhibition, working memory and task switching. The analysis of previous findings shows that dealing with goal-irrelevant, non-salient information received far more attention than generalizing in the presence of goal-irrelevant, salient information. The developmental timing of the latter can only be inferred from few transfer studies, as well as executive functions, planning and theory of mind research, to highlight gaps in knowledge and sketch out future research directions. Understanding how transfer in the presence of seemingly relevant but truly irrelevant information develops has implications for well-balanced participation in information societies, early and lifespan education, and investigating the evolutionary trajectory of flexible problem solving.

## 1. Introduction

Transferring knowledge across situations is a pivotal skill in dynamically changing environments that humans inhabit and shape. This skill supports generalizing a response to a single situation across other, unfamiliar but equivalent situations and, thereby, facilitates efficient problem-solving (e.g., Gray and Holyoak 2021; Gentner et al. 2003, 2004).

Generalizing responses across unfamiliar situations draws on previously acquired knowledge and supports tailoring familiar solutions to unfamiliar problems. This capacity is central to human problem solving in that it boosts flexibility of individual behavior. With the access to such generalization, individuals can accurately respond to never-seen situations that only partially overlap with previously encountered ones. Generalization of previous knowledge, therefore, underpins human flexible problem solving. Flexible problem solving begins to develop early, in the first year of life, and markedly improves throughout toddlerhood and the preschool years. Increasingly efficient generalization of knowledge across situations, that underpins such development, has been repeatedly investigated in the last decades within separate but interconnected areas of developmental research on memory, executive functions, planning and theory of mind. However, a unifying perspective on these findings, accounting for the role of the salience of goal-irrelevant information involved in transfer, is currently lacking, obscuring the central role of flexible problem solving in child’s everyday functioning and education. Flexible problem solving draws on long-term memory retrieval (Gentner et al. 2001; Holyoak and Thagard 1997; Hummel and Holyoak 2002; Kanerva 1988; Kokinov 1988) and executive functions, i.e., top-down processes that support handling retrieved information alongside information incoming from the environment in real-time (e.g., Zelazo et al. 1997). Therefore, in this review paper, previous findings on the interplay of early problem-solving, memory and executive functions are gathered, organized, and integrated to highlight how memory and executive functions support flexible problem solving and delineate gaps in the current state of knowledge. This, in turn, may further the current understanding of the development and the significance of flexible problem solving and support sketching out future research directions. Considering how memory and executive functions support flexible problem solving, will prospectively allow for integrating findings from developmental psychology and animal cognition.

## 2. Flexible Problem Solving

The term of flexible problem solving is not new in cognitive research and was repeatedly used either in the context of cognitive flexibility^1^ (also termed set-shifting or taskswitching), a core executive function defined as the ability to flexibly switch between different task dimensions (e.g., Chevalier and Blaye 2008; Cragg and Chevalier 2012; Deák 2000; Espy 1997; Espy and Cwik 2004; Frye et al. 1995; Jacques and Zelazo 2001; Smidts et al. 2004; Zelazo et al. 1996, 2003), or in relation to changing task demands, requiring the individual to abandon a previously applicable solution after a change in the task structure (Hopper et al. 2020). In the first case, the task remains the same, but the individual needs to shift attention to another aspect of the task. In the second case, the task changes, and the individual needs to spot the change and act accordingly. The first use, in the context of cognitive flexibility, is prevalent in developmental psychological research, and the latter, in the context of the changing task demands, is more common among comparative psychologists interested in human development. For example, the term of flexible problem solving in the developmental research was used to denote cognitive capacities tested in card-sorting tasks, such as the Dimensional Change Card Sort in which children typically need to switch between acting upon a color or a shape of an object presented on a series of cards (DCCS; Frye et al. 1995; Zelazo 2006) or the Pattern Completion Task, in which children need to detect and switch between rules applied to a pattern of objects and choose a correct object to complete the pattern (PCT, Bennett and Müller 2010). Conversely, flexible problem solving in animal cognition research was measured, for instance, with a physical task that required great apes and children to retrieve a ball from inside of a clear tube by removing straws supporting the ball (Hopper et al. 2020). Over the course of the task, the configuration of the straws changed, and demanded reassessing the situation and switching to another solution.

Using the term of flexible problem solving in the two presented ways limits the potential of the term, which, thanks to the “problem solving” component encompasses a broad range of problem-solving paradigms, and thanks to the “flexible” component can narrow this range to paradigms in which individuals need to deal with currently goal-irrelevant information that, however, may have been goal-relevant in previous, similar situations. Therefore, in this literature review, flexible problem solving is defined as a comprehensive term encompassing processes that share a common objective of managing information from the environment or long-term memory. These processes involve selecting goal-relevant information while disregarding goal-irrelevant information, even if it may have been relevant in previous situations. This allows for including diverse paradigms, such as deferred imitation, preferential looking, physical problem solving, object search and A:B::C:D, since all these paradigms involve manipulating information goal-relevance. For instance, in deferred imitation, children are encouraged to repeat a sequence of actions with a set of props, previously demonstrated by the experimenter. The physical appearance of the props might vary across sessions in terms of shape and color. Crucially, these props were earlier associated with one action and, thus, could have been associated with this task , but are, in fact, goal-irrelevant. Thus, in later encounters of the task, they should be dissociated from the task (flexibility). The sequence of actions is goal-relevant and should be imitated by a child regardless of the goal-irrelevant changes in shape or color (e.g., Hayne et al. 1997).

The processes that serve the same broad function, of dealing with currently goal-irrelevant information that, however, may have been goal-relevant in previous, similar situations, were repeatedly investigated in developmental psychological research under the terms of representational flexibility, memory generalization, analogical transfer, and, as mentioned above, cognitive flexibility, but, so far, their common function has not been at the forefront of this research, hindering integrative outlooks on the findings generated separately under each of these terms. Definitions of representational flexibility, memory generalization, analogical transfer and cognitive flexibility diverge (Table 1) but share a common denominator in that all involve acting upon previously acquired knowledge in the face of a partly unfamiliar situation. Furthermore, all these definitions draw on executive functions and all, except for cognitive flexibility, explicitly draw on long-term memory.

### 2.1. Memory, Executive Functions and Transfer

Dealing with currently goal-irrelevant information that, however, may have been goal-relevant in previous, similar situations draws on long-term memory (e.g., Chen et al. 2004; Gentner et al. 2004) and cognitive control (executive functions; EFs), a set of top-down processes that facilitate efficient control over information processing (Diamond 2012; Glady et al. 2017; Miller and Cohen 2001; Thibaut and French 2016; Richland and Burchinal 2013). Although multiple accounts of how long-term memory and executive functions separately underpin flexible behaviors have been developed (long-term memory: Chen et al. 2004; Gentner et al. 2004; executive functions: Glady et al. 2017; Thibaut and French 2016; Richland and Burchinal 2013), we propose that accounts focusing on both these capacities and their interplay would be most productive in this literature review. According to such accounts, memory systems have evolved to enable individuals to draw on past experiences when solving never-encountered yet partially overlapping problems (Bobrowicz 2019; Kanerva 1988; Lee 2009; Robertson 2012). A behavioral response to such a never-encountered problem is preceded by a retrieval of partly overlapping memories from long-term memory paired with inhibition of competing memory traces (Anderson 2003; Anderson et al. 1994, 2004; Bekinschtein et al. 2018; Snyder et al. 2014).

In the face of the never-encountered problem, individuals can rely on memories that only partly overlap with the problem at hand because potentially overlapping features of the problem cue and activate several memory traces, matching some features of the retrieval cue (Anderson and Levy 2009; Tulving 1974; Tulving and Pearlstone 1966). The activation of multiple, potentially relevant memory traces results in retrieval competition. Thereafter, the most goal-relevant trace is selected through inhibition of the other, competing traces; such inhibition, responsible for adaptive forgetting of competing memories (Anderson 2003; Anderson et al. 2004) belongs to the executive function repertoire (Johansson et al. 2007; Peters et al. 2013).

Long-term memory processes supply the individual with relevant information on previous, partly overlapping encounters, but cognitive control over memory retrieval needs to be paired with cognitive control over attention and working memory before issuing the behavioural response (e.g., Howard et al. 2014; van Moorselaar and Slagter 2020). From a problem-solving perspective, the problem at hand is essentially an arrangement of features that vary in salience and relevance for the solution. The individual needs to attend to the potentially goal-relevant information, hold the goal-relevant information temporarily in working memory and compare it with traces retrieved from long-term memory.

Memory retrieval, triggered by the cues in the novel situation, results in a pool of potentially applicable memory traces but does not specify which of these traces should be applied in the novel situation. Therefore, generalizing knowledge across situations, that demand inhibiting goal-irrelevant information, must draw on executive control. The retrieved information must be sorted, selected, and prioritized by executive functions (EFs), a set of top-down cognitive processes that exert intentional control over all information processing, from acquiring the information from the environment to issuing a behavioral response (Bobrowicz 2019). EFs allow coordinating attention and action and thereby underpin the three steps that support generalization of knowledge (Diamond 2012; Miller and Cohen 2001): (1) suppressing irrelevant pieces of information, (2) holding the relevant information in working memory, and (3) switching between relevant bits of information to identify the most relevant ones. According to one of the most common conceptualizations of EFs, these three tasks match three core EFs: executive inhibition, working memory and task switching, also termed set-shifting or cognitive flexibility (Diamond 2012; Miyake et al. 2000; Nigg 2000; Wiebe et al. 2010). However, understanding how the core EFs underlie memory generalization is not possible without understanding how attention, working memory and long-term memory are related. According to Baddeley’s model (Baddeley 2000; preceded by Baddeley and Hitch 1974), the central executive, a part of the working memory system, acts as a meeting point between information from the environment and information retrieved from memory. Conceptualizing working memory as an intersection of information available in attention and long-term memory is key to other memory models, too (e.g., Oberauer 2002; Pascual-Leone and Johnson 2005).

Executive inhibition involves both memory inhibition and attentional inhibition (e.g., Howard et al. 2014; van Moorselaar and Slagter 2020). Attentional inhibition (also: selective attention) supports overriding attention to salient perceptual similarities in favor of functional ones (Simms et al. 2018; Thibaut and French 2016) and disregarding immediate salient, yet irrelevant associations (Thibaut and French 2016). Memory inhibition supports suppressing competing yet currently irrelevant traces in long-term memory and preventing them from entering working memory (e.g., Anderson 2003; Anderson and Levy 2009; Bobrowicz 2019; Nigg 2000). Such memory inhibition will be discussed in relation to complex transfers that demand inhibiting salient yet goal-irrelevant information.

Thanks to both memory and attentional inhibitory processes, goal-irrelevant information is kept out of working memory that holds potentially goal-relevant information “online”, either acquired from the environment or retrieved from long-term memory (Baddeley and Hitch 1974; Diamond 2012; Smith and Jonides 1999). Therefore, working memory supports extracting features from objects, detecting relationships between such features (Diamond 2012) and combining information acquired in the present with that acquired in the past. Inhibition and working memory are, together, responsible for sorting and selecting goal-relevant information that must be prioritized by task-switching EF processes, responsible for shifting between potentially goal-relevant information bits and selecting the most goal-relevant ones before acting upon them (Megalakaki 2016).

### 2.2. Simple and Complex Transfers

Flexible problem solving, which involves distinguishing between goal-relevant and goal-irrelevant, previously acquired information when solving unfamiliar situations, draws both on long-term memory and executive functions (Bobrowicz 2019), and weighing relevance of information lies at its core (Kanerva 1988). In principle, tailoring a solution to the problem at hand demands comparing this target problem to a pool of other, somewhat similar source problems encountered in the past, and detecting which features of these source problems are potentially relevant for the solution. Thereafter, the relevance of these features must be evaluated before issuing a response that was successful under similar circumstances. This was, for instance, investigated in a recent study with great apes (Bobrowicz et al. 2020b). The apes received a series of three problems, and their final score would depend on the final problem in the series. The apes were confronted with a final test problem, after receiving two other problems: one that demanded the same action but looked different, and another that required a different action but looked the same as the final test problem. The action served as the goal-relevant information, contrary to the physical appearance that served as the goal-irrelevant information. In this setup, a solution that was previously applicable to a superficially identical problem would no longer work in the final problem, and so had to be discarded in favor of the solution acquired on a superficially different but in fact goal-relevant problem (Bobrowicz et al. 2020b).

Both relevance and similarity of information are context-dependent features that change over time (Rapp and McCrudden 2018; Saracevic 2007; Soergel 2018), and just like they vary across contexts, they can be manipulated across experimental conditions (e.g., in narrative tasks: Rapp and Gerrig 2006; Rapp and McCrudden 2018; in analogical reasoning tasks: Glady et al. 2017; Thibaut et al. 2010; Richland et al. 2006). Consider the following example. In an analogical reasoning task (Glady et al. 2017), children were presented with two related items, A and B (e.g., a bird and a nest), and were supposed to uncover and use this relation to find a match for item C (a dog) in a pool of potential Ds (a doghouse, a bone, an apple and a guitar). Two experimental conditions were introduced, one in grayscale, and another in color. In both experimental conditions, children’s attention was drawn to the A:B pair, but children’s results depended on whether the most salient relation between A and B (colour) was irrelevant for the C:D pairing (which should have relied on a semantic match). These examples show that (1) individuals can be supplied with irrelevant information over the course of an experiment, and (2) that experimentally manipulated salience of information can hinder assessment of information relevance.

Finding that salient yet irrelevant information can hinder flexible problem solving is consistent with neuropsychological evidence showing that inhibition of competing memories depends on the salience and relevance of the retrieved information (Snyder et al. 2014). Although goal-relevance is the key feature of the cued traces that guides retrieval, some of goal-irrelevant traces may become activated alongside the goal-relevant traces, if they are salient enough. For instance, imagine that you once used a yellow key with five cuts in the key blade to open a rarely used door in the past. If you need to open the door again, holding a keychain with two keys, a yellow and a blue one, you might be more likely attempt opening the door with the yellow key, ignoring the number of cuts on the present keys. Even if the blue key has a five-cut blade and even though you well know that the shape of the blade, and not its color, determine its usability, you may act on the more salient feature of the present situation. In this case, salience of each key was incongruent with its relevance for the goal.

The adult prefrontal cortex can deal with most cases of memory competition and act upon goal-relevant traces even if salient, yet goal-irrelevant traces are activated, too (Snyder et al. 2014). Although this ability is key for flexible problem-solving, it has been overlooked in developmental psychological research, which focused rather on the retrieval of a single goal-relevant memory or resolution of the so-called underdetermined competition, wherein several goal-relevant memories competed for retrieval (e.g., Crisafi and Brown 1986). In this review, tasks that hint on the development of such retrieval are called *simple transfer* tasks (see below). Resolution of the so-called prepotent competition, in which a strongly salient yet goal-irrelevant memory is involved alongside a less salient yet goal-relevant memory, were studied to a far less extent. Tasks that hint on how resolution of the prepotent competition develops are called *complex transfer* tasks in this review (see below).

Simple transfer tasks, measuring generalization of knowledge across partially similar problems, typically involve two problems: a source and a target (e.g., Chen 1996). Whereas the source and the target are typically perceptually different, they share a common principle for solution, that is, they are functionally equivalent. Retrieval of the source will provide goal-relevant information for the solution of the target. For instance, in a source problem, one may learn how to use a touchpad, sliding the tip of the finger across its surface and coordinating this movement with the cursor’s movement on screen. This link between the movements of the finger and the cursor may be later applied to a touchscreen, that is, a different-looking target problem that likewise requires a coordination of such movements.

In everyday life, individuals solve various problems that contain goal-irrelevant information between the source and the target. This information can either be perceptually dissimilar to the target (weak salience) or be perceptually similar to the target (strong salience). In the case of weak salience, the goal-irrelevant information will rather not compete for retrieval. In the case of strong salience, the goal-irrelevant information will become activated alongside the source, engaging in prepotent competition for retrieval (Snyder et al. 2014). Note that transfer tasks that involve several goal-relevant source information preceding the target problem, potentially involved in the underdetermined competition, are also classified as simple transfers in this review. Therefore, Crisafi and Brown’s task that required integrating relevant information and ignoring distracting information acquired on two separate source problems (Crisafi and Brown 1986) is classified as a simple transfer task. How simple transfers are mastered in development was repeatedly investigated in the past (Alexander et al. 1989; Bauer and Dow 1994; Barr et al. 2020; Bechtel et al. 2013; Bobrowicz et al. 2020a, 2022; Booth and Waxman 2002; Brito and Barr 2014; Brown and Kane 1988; Brown et al. 1989; Chen 1996; Chen and Daehler 1989; Chen et al. 1997; Crisafi and Brown 1986; DeLoache et al. 1991, 1999, 2004; Gentner and Markman 1997; Goswami 1989; Goswami and Brown 1989; Hanna and Meltzoff 1993; Hayne et al. 1997, 2000; Herbert 2011; Herbert and Hayne 2000; Herbert et al. 2007; Hewson 1978; Holyoak et al. 1984; Kingo and Krøjgaard 2013; Learmonth et al. 2004; Madole et al. 1993; Madole and Cohen 1995; Pauen and Bechtel-Kuehne 2016; Simcock et al. 2011; Sternberg and Rifkin 1979; Taylor et al. 2016; Thibaut and French 2016; Träuble and Pauen 2007; see Section 3 and Table 2). Simple transfers seem to be available to children as young as 6 months. Simple transfers were investigated in (1) the deferred imitation paradigm, where the child would imitate a previously observed sequence of actions; (2) preferential looking paradigm, where the child would categorize objects as similar (no change in looking time) or dissimilar (change in looking time); (3) object search tasks, where the child would use knowledge of where something in a small-scale model of a room to look for the same item in a real-life full-size room; (4) physical problem solving, where the child would use previously acquired knowledge of tool use to solve novel problems; (5) conceptual problem solving, where the child would use previously acquired relational knowledge to solve biology puzzles; (6) structure mapping tasks, where the child would use previously acquired relational knowledge to solve spatial metaphors; and (7) A:B::C:D tasks, where the child would recognize the semantic relation between A and B to find a matching item for C in a pool of potential D items. An overview of the development of simple transfers, investigated in these paradigms, is available in Table 2.

Complex transfer tasks, contrary to simple transfer tasks, involve associating strong salience with goal-irrelevance. Back to the above example, consider completing another source problem in between using the touchpad and the touchscreen. This additional source problem would involve using a computer mouse to move the cursor on a monitor that looks exactly like the above-mentioned touchscreen. This source problem may disrupt performance on the touchscreen—the participant may be cued to the perceptually similar, but goal-irrelevant and so misleading experience, and will search for the mouse before eventually touching the screen. This is also a transfer task, but a complex one, in that it requires disregarding the salient, seemingly relevant features of one source in favor of the truly relevant features of the other source. Such complex transfers have been investigated to a lesser extent, as discussed in Section 4, in scene analogy tasks, where the child would need to map one or two relations across illustrations and prioritize goal-relevant relational matches over goal-irrelevant perceptual similarity, and A:B::C:D tasks (Glady et al. 2017; Thibaut et al. 2010; Richland et al. 2006).

Although the development of complex transfers has been studied far less than the development of simple transfers, this development can be inferred from task-switching, false-belief and planning studies, as outlined in Section 5. Research methods that can be particularly helpful in investigating the development of the capacity for complex transfers, along with their limitations and the limitations of the approach adopted in this review, will also be discussed in Section 5. Finally, Section 6 will focus on the significance of early flexible problem solving and conclusions of the paper.

### 2.3. Key Concepts and Variables

The development of simple transfers and complex transfers has been investigated under various labels, such as (1) analogical transfer, (2) memory flexibility, (3) representational flexibility and (4) cognitive flexibility (Table 1). (1) Analogical transfer was defined as the ability to detect common relational principles for solution across problems that share relational similarity but differ in the surface format (Brown et al. 1989; Crisafi and Brown 1986; Gentner 1988; Gentner and Smith 2013; Holyoak 2012; Goswami 1991; Goswami and Brown 1989). (2) Memory flexibility was defined as an ability to generalize information to novel situations (Barr et al. 2020, p. 2); or a balance between remembering specific features and being able to generalize that knowledge across cues and contexts (Brito and Barr 2014, p. 1157). In other words, memory flexibility involved child’s ability to generalize goal-relevant information retrieved from the existing memory representations across delayed contexts, while disregarding goal-irrelevant information present in the memory representations and in the environment. For instance, in the deferred imitation paradigm, memory flexibility supported children’s retrieval of goal-relevant information from memory, and attending to goal-relevant information on object identity (e.g., a plush toy) rather than goal-irrelevant information on its physical appearance (e.g., gray or pink color; Brito and Barr 2014). (3) Representational flexibility was defined as an inferential use of prior knowledge in new situations (Eichenbaum 1997; Hayne et al. 2000), and in fact, was conceptually identical to memory flexibility, as it allowed retention of goal-relevant information beyond immediate contexts, and its retrieval in a delayed context despite goal-irrelevant information present in both contexts. The term of representational flexibility was likewise used in the deferred imitation paradigm. According to this paradigm, representational flexibility supported children’s retrieval of goal-relevant information from memory and attending to goal-relevant information on object identity (e.g., a face or a rattle) rather than goal-irrelevant information on its physical appearance (e.g., shape or color; Hayne and Gross 2015). Finally, (4) cognitive flexibility was defined as a broader set of cognitive processes that supports analogical transfer (e.g., Richland and Morrison 2010), and encompasses memory flexibility and representational flexibility along with attentional control, working memory, inhibitory control, and set-shifting/task switching (e.g., Dajani and Uddin 2015). In a narrower definition of cognitive flexibility, adopted in most sources referred here, cognitive flexibility is equaled with set-shifting/task switching, i.e., the ability to flexibly switch between different task dimensions (e.g., Chevalier and Blaye 2008; Cragg and Chevalier 2012; Deák 2000; Espy 1997; Espy and Cwik 2004; Frye et al. 1995; Jacques and Zelazo 2001; Smidts et al. 2004; Zelazo et al. 1996; Zelazo et al. 2003). Therefore, cognitive flexibility was predominantly addressed in tasks that involved sorting a series of bidimensional stimuli, first, according to one dimension, e.g., color (red or blue), and second, according to another dimension, e.g., shape (a rabbit or a boat; Zelazo 2006). While the definitions of analogical transfer, memory/representational flexibility, and cognitive flexibility diverge, they all involve acting upon previously acquired knowledge in the face of a partly unfamiliar situation, and so potentially hint on the development of simple transfers and complex transfers.

Several variables were manipulated across studies presented in this review to reveal the developmental trajectory of simple transfers and complex transfers. Among these variables, age and delay between encoding and retrieval (or between the source and the target) were manipulated to better understand whether, with age, transferring information becomes increasingly immune to protracted delays. Richness of cues at encoding (source) and retrieval (target) was also manipulated to investigate whether, with age, transferring information becomes increasingly immune to scarcity of retrieval cues, for instance, the lack of common verbal labels provided at encoding and retrieval. Finally, growing up in monolingual vs. bi-/multilingual environments seems to be an important variable in early problem-solving, as several previous studies showed that 6- to 24-month-old bilingual children perform better at transfers than age-matched monolingual children (Barr et al. 2020). This difference may be underpinned by better-developed executive functions (Barr et al. 2020; Kovács and Mehler 2009) or rather richer encoding of the source by the bilinguals as compared to monolinguals, given the extensive critique of the EFs account (Paap et al. 2014, 2015, 2018; Paap and Sawi 2014). While the debate around the relationship between language status, EFs and transfer remains unresolved, child’s language environment remains an important variable in previous research on early problem-solving. Therefore, both studies with monolinguals and bilinguals will be presented and discussed in this review.

## 3. The Development of the Capacity for Simple Transfers

### 3.1. Early Sensitivity to Goal Relevance

Sensitivity to relations between objects and the ability to infer such relations underpins analogical reasoning and, therefore, is a precursor of simple transfers, as was recently shown in infants as young as three months (Anderson et al. 2018; also in 7- and 9-month-olds; Ferry et al. 2015). Previous findings suggest that, when inferring relations between objects, infants are sensitive to features that hint on object’s goal-relevance, e.g., shape, size, rigidity, rather than to goal-irrelevant surface features, e.g., color (Bates et al. 1980; Brito and Barr 2014). This sensitivity allows children to rapidly acquire goal-relevant knowledge on object function in the first year of life, preceding transfer of such knowledge around 12 months. In experimental setups, object function was, for instance, manipulated through a rotation of a T-shaped component, attached to a rectangular box, either pointing outwards and resembling a pin, or pointing inwards and resembling a hook (Träuble and Pauen 2007; see also Table 2). However, spontaneous, unprompted transfers of object function may remain challenging for 24-month-olds. While 24-month-olds can generalize function across objects when solving a single problem (Pauen and Bechtel-Kuehne 2016), they cannot spontaneously apply previously acquired functional knowledge to a different-looking problem in deferred imitation tasks (e.g., Herbert and Hayne 2000), object search tasks (e.g., DeLoache et al. 2004) and tool-use transfer tasks (Bobrowicz et al. 2020a; Bobrowicz et al. 2022; Chen et al. 1997; see Table 2).

### 3.2. Simple Transfers

Problem-solving tasks allow for testing not only how knowledge about objects and relations develops with age, but also how such knowledge facilitates flexible problem solving. It seems that, under certain circumstances, such generalization is available even to 6-month-olds (Brito and Barr 2014), although not monolingual and not in experimental procedures with high conceptual and motor demands. In this section, first, studies with monolingual children tackling conceptually (e.g., detecting an unobservable relation between two problems) and motorically demanding experimental procedures (e.g., grabbing a tool and applying it to a puzzle box) are presented and, thereafter, challenged by studies with mono- and bilingual children tackling less demanding experimental procedures.

Manual problem-solving tasks have been repeatedly used to investigate analogical transfer, defined as the ability to detect common principles for solution across problems (e.g., Bobrowicz et al. 2020a; Crisafi and Brown 1986). In analogical transfer, individuals need to detect a common relation between components in the source that could be mapped onto the target, disregarding surface dissimilarity between the source and the target. In other words, individuals are required to detect and transfer goal-relevant information while disregarding perceptually dissimilar and goal-irrelevant information. Crisafi and Brown (1986) suggested that examining analogical transfer in young children required “several analogical versions of the same task (i.e., sharing similarity at a deep level but differing in surface format)” (Crisafi and Brown 1986, p. 954). Detecting the relational similarity in the presence of dissimilar surface features required, in practice, detecting the common functional features and inhibiting the distracting perceptual features. This ability considerably improves between 2 and 5 years of age, when simple transfers become increasingly spontaneous and immune to a mismatch between the source and the target (Brown 1989; Brown et al. 1989; Chen 1996; Crisafi and Brown 1986; Brown and Kane 1988; Goswami 1991; Holyoak et al. 1984). At 2.5, but not earlier, children can spontaneously identify and flexibly apply relevant knowledge acquired on a functionally similar task (Bobrowicz et al. 2020a; DeLoache et al. 2004; Herbert and Hayne 2000). Even at 2, however, children transfer functional knowledge across perceptually dissimilar tasks, if the link between the source and the target is highlighted by experimenter (Crisafi and Brown 1986; Goswami 1991; Hayne and Gross 2015).

Herbert and Hayne’s deferred imitation study (Herbert and Hayne 2000), DeLoache and colleagues’ object search study (DeLoache et al. 2004), as well as Bobrowicz and colleagues’ tool-use study (Bobrowicz et al. 2020a) showed that, once children were capable of spontaneous analogical transfer, such transfer was equally efficient after a short (up to 30 min) and a long, 24-h delay (Bobrowicz et al. 2020a; DeLoache et al. 2004; Herbert and Hayne 2000). This was not surprising, given the rapid development of memory retrieval that occurs in the first two years of life (Barr et al. 1996; Hartshorn et al. 1998; Hartshorn and Rovee-Collier 1997; Hayne et al. 1997).

While sources and targets are common terms in analogical transfer research, they are conceptualized as encoding contexts and retrieval contexts, respectively, in memory research. For instance, in the deferred imitation paradigm, the situation in which the child observes the initial presentation of a sequence of actions presented by an experimenter, is called the encoding context. The situation in which the child is supposed to later reconstruct the same sequence, perhaps with different props, is called the retrieval context.

Sources and targets always belong to an idiosyncratic context, consisting of a location, time of day, and occur with other items in this context. The source serves as the encoding context, and the target serves as the retrieval context. The overlap between these contexts may vary, but they always have some common features, called cues. Transfer of knowledge across contexts demands detecting cues that would trigger the retrieval of potentially relevant features, encountered in the encoding context.

At least two terms refer to transfer across contexts in developmental research: memory flexibility (Barr and Brito 2013; Borovsky and Rovee-Collier 1990; Brito and Barr 2014; Karmiloff-Smith 1998; Learmonth et al. 2004) and representational flexibility (Eichenbaum 1997; Hayne et al. 2000), and both were investigated in so-called memory generalization tasks. Note that memory flexibility was defined as an ability to generalize information to novel situations (Barr et al. 2020, p. 2); or a balance between remembering specific features and being able to generalize that knowledge across cues and contexts (Brito and Barr 2014, p. 1157), and representational flexibility was defined as inferential use of prior knowledge in new situations, i.e., applying a previously learnt relation between two stimuli to a new situation (Eichenbaum 1997; Hayne et al. 2000; see also Table 1). Both memory flexibility and representational flexibility are assumed to rely on the same ability, that is, detecting common features in the present context and selected past contexts. Infants need to accumulate knowledge about such features across a variety of encoding contexts and apply what they learned to diverse retrieval contexts, often after considerable delays (Barr et al. 2020; Gerhardstein et al. 1998). Early in development, the features detected at encoding and at retrieval must be identical, or else infants cannot access and retrieve potentially relevant knowledge. With age, memory retrieval becomes increasingly immune to mismatches and delays between contexts, both in terms of recognition, as showed in mobile conjugate paradigm (e.g., Borovsky and Rovee-Collier 1990; Hartshorn and Rovee-Collier 1997; Hartshorn et al. 1998) and genuine recall, as showed in deferred imitation paradigm (e.g., Barr and Brito 2013; Brito and Barr 2014; Hanna and Meltzoff 1993; Hayne 2004; Hayne et al. 1997, 2000; Herbert and Hayne 2000; Learmonth et al. 2004). For instance, in deferred imitation studies, after 24 h, 12-month-olds could imitate the previously seen sequence, even if the props changed in color (e.g., gray to pink mouse) but not in shape (e.g., gray mouse to rabbit; Hayne et al. 2000). Only at 18 months, children would correctly imitate the sequence of actions with a simultaneous change in color and shape of the prop (e.g., gray mouse to pink rabbit; e.g., Brito and Barr 2014; Hayne et al. 1997), even after a considerable two-week delay (e.g., Kingo and Krøjgaard 2013). Note that a recent study (Brito and Barr 2014) somewhat challenged this developmental timing of memory generalization, finding that monolingual 6-month-olds can generalize, at least after 30 min, a sequence of actions to a novel prop that differs in color and bilingual 6-month-olds can generalize such a sequence to a novel prop that differs in both color and shape from the original prop.

Furthermore, note that flexible problem solving may actually be aided by long delays, as long as these delays include a period of sleep. Long, e.g., 4- or 24-h delays, may aid recall of information that has been consolidated in long-term memory, e.g., over a period of sleep (Konrad et al. 2016). For instance, 12-, 15- and 24-month-olds that took a nap between demonstration and imitation session in the deferred imitation paradigm show better inhibition of the irrelevant information provided during demonstration (Konrad et al. 2019). This effect was absent if children did not have a chance to take a nap. However, note that the effect of sleep on retrieval does not always occur in adults either (e.g., Davidson et al. 2020).

On the one hand, spontaneous memory generalization may develop around 6 months for some perceptual features, e.g., color and shape (deferred imitation; Brito and Barr 2014), and even around 30 months for complex situations, e.g., transferring tool use across perceptually dissimilar problems. On the other hand, constraints on memory generalization can be reduced even at 3 months by exposing infants to diverse cues and contexts at encoding (Borovsky and Rovee-Collier 1990). Encoding information in several, partially overlapping, contexts allows for accumulating knowledge about clusters of features, and thereby increases the pool of potential retrieval cues (Herbert et al. 2007; Learmonth et al. 2004). Perhaps for this reason, external verbal cues facilitate memory generalization, such as made-up “thornby” for an animal or “meewa” for a rattle, e.g., in 2-year-olds who would not otherwise be able to transfer knowledge across perceptually dissimilar contexts (Herbert and Hayne 2000). In another study, verbal labels (in English vs. Chinese, equivalents of “Look, a puppet”, “On”, “Off”, “Shake”), allowed 12-month-olds to achieve memory generalization across props of different colors and shapes that would otherwise be available only to 18-month-olds (Taylor et al. 2016). Note that providing verbal labels at encoding and retrieval did not support memory generalization across functionally equivalent props of different colors and shapes in 18-month-olds (Kingo and Krøjgaard 2013; Herbert and Hayne 2000) or even hindered memory generalization in 15-month-olds transfer of learning from 3D to 2D displays when both object names and verbs were provided (Zack et al. 2013).

Overall, verbal labels may aid memory retrieval even at 6 months because they allow the child to acquire richer information about the source, detect unobservable conceptual similarities between perceptually dissimilar contexts, and boost children’s attention at demonstration and test (e.g., Barr et al. 2020; Brito and Barr 2012; Taylor et al. 2016). Although verbal labels may help the child to notice the link between contexts, such labels may fail to facilitate performance in tasks, in which child’s cognitive capacities are not developed enough to meet the other task-specific demands or in which too many or too complex verbal labels are provided (Zack et al. 2013).

### 3.3. Memory Generalization: The Special Case of Bilingualism

Memory generalization becomes increasingly immune to changes in cues, contexts, and delays between the 6th and the 24th month of life (Taylor et al. 2016), but its developmental timing differs between monolingual and bilingual children. Bilingual children between 6 and 24 months have repeatedly performed better on memory generalization tasks that required deferred imitation than age-matched monolingual children (Barr et al. 2020), and this bilingual advantage was detected as early as at 6 months (Brito and Barr 2014). For instance, at 6 months, generalizing across props that differ both in color and shape is available to bilinguals but not monolinguals (Brito and Barr 2014). Later, at 24 months, bilinguals can spontaneously transfer knowledge across two perceptually dissimilar sets of objects, while monolinguals need a verbal label added at encoding and retrieval to do so (Barr et al. 2020; Brito et al. 2014). Further, bilinguals can benefit from receiving verbal labels at encoding and retrieval earlier in development than monolinguals, at 18 rather than 24 months in monolinguals (Barr et al. 2020). Bilinguals tend to benefit from verbal labels at encoding and retrieval earlier in development, and even when such labels are absent, they spontaneously generalize across two perceptual cues at an earlier age.

However, why bilinguals have such an advantage over monolinguals in the development of memory generalization, remains unclear (Brito and Barr 2014). It was hypothesized that, thanks to early acquisition of two languages, young bilinguals benefit from better-developed executive functions (e.g., Bialystok 1999) than age-matched monolinguals. Bilinguals maintain two “active” languages and must inhibit one when using the other, even before they can produce words in either language, and, therefore, receive more opportunities of practicing executive control than monolinguals (Bialystok 1999; Brito and Barr 2014; Kovács and Mehler 2009). Changes in executive control may, in turn, increase the efficiency of memory processing (Brito and Barr 2014) and, thereby, memory generalization. In line with this hypothesis, bilingual advantage in memory generalization at 6 months coincides with better executive control at this age in bilinguals (Kovács and Mehler 2009), compared to monolingual peers. Bilingual advantage in executive control has been repeatedly established even later in development, both in older children (e.g., Carlson and Meltzoff 2008) and adults (Bialystok 2005; Bialystok and Martin 2004).

In line with the EFs account, Crivello and colleagues (Crivello et al. 2016) found that bilinguals had superior inhibition of attention and cognitive flexibility than monolinguals at 24 and 31 months. In this longitudinal study, bilinguals outperformed monolingual peers on working memory and task-switching tasks that required suppressing attention to previously relevant, but now salient yet goal-irrelevant information (Crivello et al. 2016). It was hypothesized that task switching, increasingly practiced by bilinguals as compared to monolinguals, may boost their ability to selectively attend to, integrate and adapt to multiple cues in the environment (Barr et al. 2020; Deák and Wiseheart 2015). In line with this hypothesis, bilinguals indeed showed superior speed, i.e., better intentional inhibition, and accuracy, i.e., better task switching, than age-matched monolinguals on both set-shifting tasks (e.g., the flanker task: Costa et al. 2008; Tao et al. 2011; Yoshida et al. 2011; the Simon task: Bialystok et al. 2005; Martin-Rhee and Bialystok 2008)

Despite well-documented differences in cognitive flexibility between mono- and bilingual children (e.g., Adesope et al. 2010; Barr et al. 2020; Bialystok et al. 2005), as well as adults (Bialystok et al. 2005; Costa et al. 2008), several studies with child and adult populations failed to find such bilingual advantage (Duñabeitia et al. 2014; Esposito et al. 2013; Paap et al. 2015; Ross and Melinger 2017). These mixed results suggest that detecting the bilingual advantage may be impeded by nonoptimal task difficulty, or that bilingual advantage may be task-specific and sample-specific (Ross and Melinger 2017). Furthermore, recent metaanalyses and conceptual analyses have shown that, in both children and adults, bilingual advantage may be absent (children: Lowe et al. 2021; adults: Paap and Sawi 2014; Paap et al. 2014, 2015, 2018), and that the observed differences between mono-and bilinguals may rather result from confounding variables, such as age, language and task.

## 4. The Development of the Capacity for Complex Transfers

### 4.1. Inhibition, Working Memory, and Task Switching

Executive functions are critical to optimal cognitive development because they regulate attention and memory processes, responsible for flexible application of knowledge to novel, nonroutine situations (Bell and Cuevas 2015; Espy 2004; Diamond 2012; Miyake et al. 2000). In adults, measures of executive functions have been shown to load onto three correlated but separate factors: inhibition, working memory, and task switching, but this separability may not emerge before the school age (Wiebe and Karbach 2018), although no consensus on this issue has, so far, been achieved (Barkley et al. 2001; Best and Miller 2010; Brocki and Bohlin 2004; Isquith et al. 2004; Miyake et al. 2000). In practice, however, children improve on inhibition tasks sooner than on working memory tasks, and on working memory tasks sooner than on cognitive flexibility tasks (Blakey et al. 2016; Chevalier et al. 2012).

### 4.2. Goal-Irrelevance vs. Salience

Previous research progressively revealed that younger and younger children could transfer knowledge across contexts if, e.g., given the same verbal cue at encoding and retrieval. Even two perceptually dissimilar but functionally similar cues did not impede transfer in 6-month-old bilinguals and 12-month-old monolinguals. However, it was not until 30 months that children could spontaneously transfer goal-relevant knowledge across two more conceptually demanding, perceptually dissimilar situations where goal-relevance and salience were not pitted against one another (simple transfers), for instance, in a physical problem solving task that demanded transferring tool use across two different-looking boxes (Bobrowicz et al. 2020a, 2022).Therefore, it seems that capacity for simple transfers develops early, in the first year of life, but only at age 2.5 children become able to spontaneously detect a link between two perceptually dissimilar situations and inhibit goal-irrelevant information in favor of goal-relevant information. This draws on inhibition, sufficiently developed around age 2, and working memory, sufficiently developed around age 3 (Carlson et al. 2015). Another study, by Blakey and colleagues (Blakey et al. 2016), has also shown that children’s inhibition of goal-irrelevant non-salient information significantly improved between 2.5 and 3 years of age. While inhibiting goal-irrelevant information is a common challenge in everyday life, inhibition itself is not sufficient in situations that require switching between previously goal-relevant information that remain salient but are no longer goal-relevant. Since the ability to disregard salient but in fact goal-irrelevant information is a hallmark of complex transfers, Blakey and colleagues’ results suggest that complex transfers may be available to 3–3.5-year-olds at the earliest.

Complex transfers require inhibiting attention to previously goal-relevant but now still salient yet goal-irrelevant, misleading information, but also representing and maintaining the currently relevant goal. Since representing and maintaining the current goal may be sufficiently developed only around the age of 4, 3–3.5-year-olds may fail complex transfers. For instance, Chevalier and colleagues (Chevalier et al. 2012) reported that 4–5-year-olds were more efficient at switching than 3-year-olds in the Shape School task that required rapid naming of stimuli according to color or shape, suggesting that the older preschoolers were better at representing and maintaining the current goal than the younger ones (see also Chevalier and Blaye 2008, 2009). That goal representation and maintenance develops around 4 was further corroborated by Dietz and colleagues’ planning study (Dietz et al. 2019), in which 4-year-olds but not 3-year-olds were able to successfully evaluate feasibility of different problem-solving strategies. In a similar vein, Jacques and Zelazo showed that 4-year-olds but not 3-year olds benefitted from verbalizing the previously relevant and the currently relevant rules in task switching, as long as children formulated these rules on their own (Jacques and Zelazo 2001; as in simple transfers; Brown and Kane 1988). Prompting the child to verbalize spontaneously used rules might have strengthened representation and maintenance of the currently relevant goal, which, according to previous findings, improves after the age of 4 (as shown in another, complex transfer task; Glady et al. 2017). The improvement in goal representation and maintenance continues between age 4 and 6, resulting in a rapid development of children’s planning abilities (Gardner and Rogoff 1990; Klahr and Robinson 1981; Tecwyn et al. 2014).

Overall, previous findings suggest that improvements in coordination of inhibition and working memory, goal representation and conceptual knowledge all play a role in switching between potentially relevant information bits (Blaye and Jacques 2009). Goal representation improves significantly between 3 and 4 years of age, and coordination of inhibition and working memory significantly improves between 4 and 6 years of age (e.g., Chevalier and Blaye 2008, 2009; Chevalier et al. 2012; Zelazo 2006). Therefore, navigating between previously and currently relevant information may be available to 4-year-olds at the earliest, but only to 5–6-year-olds if the coordination of inhibition and working memory is critical to such navigation.

### 4.3. Complex Transfers

Switching tasks involve a pool of items, but these items are usually presented sequentially. This removes potential distraction and conflict caused by other, simultaneously presented items, and therefore offers insight into child’s switching in the absence of such competitors. Switching tasks typically require inhibition of the previous rule and concentrating on the current rule, often within the same pool of items. In one such switching task, the Dimensional Change Card Sorting task (Zelazo 2006), children sort a pool of cards depicting red rabbits, blue rabbits, red boats and blue boats. First, children are asked to sort these items by color and thereafter by shape. This requires inhibiting the previous rule, keeping the current rule in mind and applying it to the presented stimuli. In the beginning, it is about color, then about shape, or the reverse, so the task does not require holding “online” two rules and switching between them.

Maintaining and switching between two rules is introduced in a more advanced version of the task (Zelazo 2006). Whenever the item is surrounded by a black border, it should be sorted by color; otherwise, it should be sorted by shape. Given the previously discussed findings, it is perhaps not surprising that 4-year-olds pass the simpler version of the task, but typically fail the advanced Border version (Zelazo 2006). Maintaining and switching between two rules is difficult even for many 5-year-olds, but not 6-year-olds. Therefore, it seems that representing and switching between two action plans may develop between 5 and 6 years of age.

How this ability develops, has been further investigated in analogy-making tasks that required inhibiting competing, seemingly relevant, perceptual matches in favor of truly relevant, functional matches (e.g., scene analogy in Richland et al. 2006; A:B::C:D in Glady et al. 2017; Thibaut et al. 2010). In a standard A:B::C:D task, used to study analogy-making, children need to detect how A is related to B and then, from among a pool of items, select D that is related to C in the same way. This requires detecting and transferring an abstract rule across two pairs of items, e.g., A (shirt) fits in B (suitcase), C (a toy car) fits in D (a box; Thibaut and French 2016). The pool of potential D items may contain the functionally relevant item (the correct D) alongside irrelevant, perceptually dissimilar items as well as irrelevant, perceptually similar items (the incorrect Ds). This allows for pitting perceptual similarity against functional similarity among the D items.

With age, children’s performance on A:B::C:D improves, but even 5- and 6-year-olds seem to misunderstand the key task rules (Thibaut et al. 2010; Thibaut and French 2016). Eight-year-olds understand the rules but suffer from competition between perceptual and functional similarities, contrary to 14-year-olds (Thibaut et al. 2010). This shows that resolving competition between perceptual and functional relationships may emerge around 8 and continue to develop in teenage years (see also Richland et al. 2006).

Although coordination of inhibition and working memory at 5–6 years may suffice for generalization when competitors are absent in the visual field, it may require further development to support generalization in the presence of such competitors (Glady et al. 2017). Increases in knowledge may also support such generalization, but it is unlikely that only 14-year-olds would have conceptual knowledge sufficient for transferring simple relations, such as “X fits in Y” across simple items that differ in colors and shapes. Eight-year-olds’ disadvantage may instead result from poorer use of executive functions than 14-year-olds’ (Thibaut et al. 2011; Thibaut and French 2016). Thibaut and French (2016) showed that 8-year-olds focus more on the C item and less on the A:B pair than adults, suggesting that, compared to 14-year-olds, 8-year-olds may have greater difficulties in inhibiting the ultimate goal of the task (studying C to find the correct D) in favor of the currently relevant subgoals (studying the relationship between A and B; drawing the relationship between C and potential Ds), and switching between the ultimate goal and the subgoals (see also French et al. 2017 for a modelling approach).

Given the previous findings, the capacity for complex transfers develops considerably in the preschool years and continues to develop in adolescence. However, it is unclear when children or adolescents develop the ability to prioritize information retrieved from long-term memory, in which functional relevance is pitted against perceptual similarity. Such information enters working memory, where potential matches are held, manipulated, and compared against the target information available in the visual field. This requires switching between the representation of the target problem and at least two representations of the source problems. Maintaining and switching between these three representations in development remain understudied. However, the developmental timing of complex transfers that require such operations can be inferred from both above-mentioned results of switching and analogy-making tasks, as well as investigations of Theory of Mind (ToM). Since performance on some ToM tasks correlates with task switching (Carlson et al. 2014, 2015) and is superior in bilinguals compared to monolinguals (Greenberg et al. 2013; Kovács 2009), this may be a good lead in estimating the developmental timing of such complex transfers.

## 5. Prioritizing Truly Relevant over Seemingly Relevant Information Retrieved from Memory

### 5.1. Switching between Representations in False-Belief Tasks

In the first year of life, as discussed in Section 3, infants rapidly gain knowledge about objects and their features. Namely, infants rapidly learn that objects are inanimate: they can be thrown and pushed but will not move on their own, without an external impulse (e.g., Luo and Baillargeon 2005; Spelke et al. 1995). This impulse can be provided only by agents, that is, active, animate entities in the environment that put objects into motion. Therefore, as infants gain physical knowledge about objects, they also gain social knowledge about agents. Even 6-month-olds expect that agents (a self-propelled box) but not objects (an inert box) can, for instance, reverse direction spontaneously, remain stationary when hit or pulled, and remain stable without an adequate support (Luo et al. 2009). Further, by the end of the first year, infants understand that agents but not objects have intentions (Baldwin et al. 2001), can track experiences of another agent and even recognize that these experiences are different from their own (Tomasello and Haberl 2003). This early intuition that others may have own desires, beliefs and knowledge underpins understanding that others hold representations and misrepresentations of reality and supports switching between own and others’ representations later, in the preschool years (Wellman and Liu 2004). The ability to understand and switch between own and others’, past and present representations of reality is termed Theory of Mind (Astington and Gopnik 1991; Baron-Cohen et al. 1985; Flavell et al. 1983). This ability has been repeatedly investigated in false-belief tasks that involved an unexpected location, unexpected contents or mismatches between appearance and reality.

The unexpected location tasks, e.g., the Sally-Anne Test (Baron-Cohen et al. 1985) or the Maxi-Task (Wimmer and Perner 1983), demand switching between two present representations: one’s own and the agent’s. The unexpected contents tasks, e.g., the Smarties Task (Astington and Gopnik 1991; Frye et al. 1995) or the Crayons Task (Hogrefe et al. 1986), tests not only whether the child can switch between their own and the agent’s representation of the present, but also whether she can switch between her own past and present representation of reality. Finally, the appearance-reality task (Flavell et al. 1983) likewise requires switching between own present and past representations of reality, but also inhibiting salient misleading perceptual features in favor of the object’s functional features in the present.

All these false-belief tasks require maintaining and switching between conflicting representations, own and others’, present and past. Regardless of who holds these representations (the child or the agent) or at what point in time she holds them (in the past or in the present), a switch in children’s performance occurs around 4 years. Three-year-olds would typically fail the language-based false-belief tasks that are listed above, answering the questions in line with their own, present representation. Four-year-olds would, on the other hand, consider and switch between own and others’, past and present representations, showing that they understand the difference between reality and representation of reality. Although some aspects of ToM, that allow for attempting lying and deception develop before 4 (Lee 2013), a vast majority of ToM studies suggests that only 4-year-olds are cognitively ready for complex transfers of knowledge. More recently, however, it was shown that even 15-month-olds could pass false-belief tasks in a non-verbal experimental procedure (Onishi and Baillargeon 2005; further discussed below).

Preschoolers’ performance on false-belief tasks that involve maintaining and switching between conflicting representations suggests that only 4-year-olds would perform complex transfers that demand inhibiting misleading information. First, executive control responsible for maintaining and switching between representations held in working memory may be underdeveloped in younger children. Second, younger children may not realize that what is held in working memory exists separately from reality and may misrepresent this reality (Onishi and Baillargeon 2005). On the other hand, even younger children can pass tasks that follow nonverbal scenarios equivalent to the verbal false-belief tasks, in which the agent misses a change of object location and mistakenly searches for it in the initial location (Onishi and Baillargeon 2005). This suggests that capacity for complex transfers could potentially be found in younger children than hitherto tested as long as the procedure would remain nonverbal. For instance, using a nonverbal violation-of-expectation task, Onishi and Baillargeon (2005) showed that even 15-month-olds expected the agent to search for the object in a location where she believed the object to be and looked longer when she did not. This suggests that even between 12 and 18 months children have a rudimentary understanding that beliefs of others may match or mismatch the reality or understand others’ beliefs but have difficulties to recode them verbally (see also Buttelmann et al. 2009).

Taken together, the reviewed findings show that inhibiting conflicting, misleading, perceptually salient information in favor of functional information should be available only to 5–6-year-olds or even 8-year-olds (Thibaut et al. 2010). However, using age-appropriate, nonverbal experimental procedures has repeatedly revealed that supposedly complex cognitive capacities may, in a rudimentary but sufficient, age-appropriate form, develop much earlier than previously thought. Therefore, investigating when and how the capacity for complex transfers emerges in development requires an inclusive nonverbal experimental procedure that could be tested with infants, toddlers and preschoolers, and even older children and adults. Since conceptual and motor demands cannot be too high in such experimental procedures, perhaps they should draw on looking-time measures.

### 5.2. Methodological Considerations

Gaze behaviors are a promising source of information on cognitive development, including the development of flexible problem solving because, they can be gathered across ages, and do not exclude young children with poor motor control and eye-hand coordination. Looking-time measures have often been used in psychological research to investigate the early and lifespan development of cognitive capacities (e.g., Baillargeon 2004; Eckstein et al. 2017; Krøjgaard et al. 2020; Onishi and Baillargeon 2005; Thibaut et al. 2011). Looking-time measures may be collected even in the first days of life, offering early insight into developmental changes in attention. In the first two months, looking-time measures are not, however, a reliable measure of infant attention, as fixations of eyes are involuntary and guided by the objects’ perceptual salience in the environment (Ruff and Rothbart 2010). Only around the 4th month, infants stop demonstrating obligatory fixations, in which they keep looking at objects, even if they are no longer paying attention (e.g., Bronson 1994; Hunnius et al. 2008). Therefore, whether infants indeed attend to the objects or not may not be reliably inferred from looking behaviors before the 4th month. However, even in newborns, attending to objects can be inferred from physiological measures, such as heart rate and respiratory sinus arrythmia. Across the life span, these measures can reliably show whether participants are actively attending to stimuli or, for instance, mind-wandering instead (Reynolds and Richards 2007; Richards 1985; Richards and Casey 1992; Ruff and Rothbart 2010). Another measure that may reveal whether participants are attentively looking at the displayed stimuli is pupil dilation (Eckstein et al. 2017). Pupil dilation is modulated by brain structures that control physiological arousal and attention and, as such, can be used as a measure of cognitive effort and task difficulty across problem-solving tasks (e.g., Beatty 1982; Boersma et al. 1970; Chevalier et al. 2012; Eckstein et al. 2017; Krøjgaard et al. 2020; Sonne et al. 2016b), in both children and adults. Increased pupil dilation correlates with increased subjective task difficulty and cognitive effort and has recently been shown in adults during a proactive interference task (Johansson et al. 2018), in which goal-relevant information competed with goal-irrelevant information in working memory. Therefore, pupil dilation may be another good measure of attention and working memory processes that support inhibiting not only distracting, but also competing and misleading information in nonverbal experimental procedures.

Although looking behaviors have been repeatedly measured in developmental psychological paradigms, some of these paradigms such as the anticipatory looking paradigm, may be more appropriate when investigating the development of flexible problem solving than others, such as the visual paired comparison paradigm and the violation-of-expectation paradigm. In the visual paired comparison paradigm (Sokolov 1963; Sonne et al. 2018), it is assumed that presentation of a novel stimulus alongside a familiar one will elicit an orienting response, draw individual’s attention, and result in longer looking time at the novel as compared to the familiar stimulus. However, some studies showed that infants (e.g., Wilk et al. 2001) and toddlers may actually look longer at the familiar stimuli in this paradigm (e.g., Hayne et al. 2016), and a lack of difference in looking time can be taken as valid evidence of recall (Hayne et al. 2016; Sonne et al. 2016a, 2018; Bahrick and Pickens 1995), showing that the delay between encoding and retrieval can be a confounding factor in interpretation of child’s performance. This can be particularly problematic in experiments that involve, for instance, more than one source problem, since, at retrieval, it can lead to overshadowing the effect of interaction (interference or competition) between the source problems by the effect of the delay on the observable result. The violation-of-expectation paradigm may be, in principle, a better alternative, as evidenced by Baillargeon and colleagues’ multiple studies on conceptual development (e.g., Baillargeon 2004; Hespos and Baillargeon 2001; Luo and Baillargeon 2005), but, on the other hand, may be prone to another confounding factor, that is, the varying degree of stimuli familiarity (e.g., Munakata 2000). Moreover, given that complex transfers involve misleading information, associated with another outcome than before, such transfers would necessarily evoke violation of expectation. This would only confirm that the child indeed recognized the current outcome as incongruent with the expected outcome but would not show whether and how the child could disregard misleading, seemingly relevant in favor of truly relevant information. Therefore, the anticipatory looking paradigm may be the best alternative out of the three, although future studies might reveal its shortcomings.

In the anticipatory looking paradigm, a participant, regardless of their age, supposedly anticipates an observed agent to act in a certain way and in a certain location and looks towards this location soon before the agent does so (Krupenye et al. 2016; Marticorena et al. 2011; Southgate et al. 2007). This paradigm has been successfully used in simplified false-belief tasks analogous to that introduced by Onishi and Baillargeon (2005), in both children (Southgate et al. 2007) and non-human primates (Marticorena et al. 2011; Krupenye et al. 2016). The anticipatory-looking tasks (as well as violation-of-expectation tasks) involve lifelike, dynamic and animated events (as in e.g., Bahrick and Pickens 1995; Kingo et al. 2014; Kirkorian et al. 2016; Sonne et al. 2018), and may therefore act as more accurate measures of cognitive development than static patterns, photos, faces and objects (but see Holleman et al. 2020). Therefore, the anticipatory looking paradigm may be a promising choice for future investigations of the capacity for complex transfers in young children. This could offer insight into both developmental and comparative trajectories of this capacity.

Measuring looking behaviors across ages in the anticipatory looking paradigm requires eye tracking and considering diverse gaze metrics (Eckstein et al. 2017), depending, of course, on specific research questions and stimuli used in a given project (Holmqvist et al. 2011). Fixations and scan paths are among gaze metrics that could be particularly helpful in measuring whether children anticipate the agent to attend to certain objects in a given location, as fixations allow for calculating time spent looking at a given location (Eckstein et al. 2017) and scan paths allow for measuring how complex stimuli, such as inanimate scenes or animated video recordings are being scanned during the experiment. Both these measures were previously used to study memory (e.g., Hannula et al. 2010; Johansson et al. 2018), problem solving (e.g., Grant and Spivey 2003) and reasoning (e.g., French et al. 2017; Thibaut et al. 2010; Thibaut and French 2016) and may likewise prove useful in future nonverbal studies of complex transfers.

### 5.3. Limitations

The approach adopted in this review offers a framework that allows for taking stock of findings from different areas of developmental research and revealing gaps in the current state of knowledge. Considering how cognitive processes are implicated in problem solving and what different problems demand from such processing is an important step in establishing that flexible problem solving is central to everyday functioning and, as such, should be supported in early education. However, the functional approach has certain limitations. Introducing flexible problem solving as an umbrella term for simple and complex transfers may be somewhat confusing. “Flexible” encompasses two different types of flexibility, in the presence of distracting information in simple transfers and in the presence of conflicting, misleading information in complex transfers. As discussed above, these two types of flexibility have distinct developmental timing and are measured in distinct experimental procedures. However, both simple and complex transfers draw on executive functions that permit flexible behaviors supported by simple and complex transfers, and, therefore, they call for a higher-level, integrative label of “flexible” problem solving. The term of “flexible problem solving” is an umbrella term that encompasses distinct behaviors measured in distinct methods yet sharing the same common demand of dealing with currently goal-irrelevant information that, however, may have been goal-relevant in previous, similar situations.

Another approach to flexible problem solving could be considered. On the one hand, knowledge about the early and lifespan development of simple and complex transfers was accumulated decade after decade, so insights into such transfers could be presented decade-wise. On the other hand, complex transfers employed in analogical reasoning were already considered in antiquity (Goswami 1991; Pellegrino 1985) but started to draw researchers’ attention only in 1980s and would be systematically studied even later, in 2000s (e.g., Richland et al. 2006; Thibaut et al. 2011; Zelazo 2006). In principle, the historical approach, outlining how philosophers’ and psychologists’ interest in transfers of knowledge unfolded over time, could have been adopted in this review paper. However, adopting this approach would likely be less productive than the functional approach. After all, it is the common function that binds simple and complex transfers and makes them an important area of future research.

Furthermore, in the approach adopted in this review, the role of language in knowledge transfers has been neglected. Verbal skills have been repeatedly found to interact with EFs in monolingual children and adults (e.g., Baddeley et al. 2001; Carlson and Moses 2001; Chevalier et al. 2012), supporting simple transfers and perhaps playing a central role in complex transfers. While verbalization was repeatedly investigated as an important factor in simple (e.g., Christie and Gentner 2014; Gentner 1977) and complex transfers (e.g., Glady et al. 2017), interactions between self-regulatory function of language and flexible problem solving call for attention in future reviews and empirical reports. For instance, in the future, it would be interesting to investigate whether self-regulatory speech interacts with complex transfers, since it has been repeatedly shown to facilitate performance in difficult planning and problem-solving tasks (Sturn and Johnston 1999; Vygotsky 1987; Winsler and Naglieri 2003). Interestingly, an increase in self-regulatory speech occurs between 2 and 5 years of age (Berk and Spuhl 1995; Furrow 1984) when children’s executive functions improve but do not allow for complex transfers, and a decrease in self-regulatory speech begins around 5–6 years (Aziz et al. 2017; Winsler and Naglieri 2003), perhaps coinciding with the onset of the capacity for complex transfers.

Nonverbal experimental procedures allow for testing how participants at different ages and even of different species perform on equivalent tasks, and, to a certain extent, allow for tracing the developmental and the evolutionary trajectories of cognitive capacities. However, even if, e.g., a preschooler and an adult chimpanzee perform on similar levels, extreme caution is needed when comparing and interpreting these results in terms of cognitive processes involved. Not only success rates, but also error patterns should be analyzed in such comparisons, and batteries of tasks, instead of single tasks, should be used.

Importantly, the developmental methods that focus on looking behaviors often cannot be uniformly tested with children between 12 and 36 months. For instance, Anderson and Levin showed that between 12 and 48 months, children look increasingly longer at animated recordings, with a sharp increase in frequency of looking at such recordings around 30 months (Anderson and Levin 1976). This finding, corroborated by other studies (Anderson et al. 1981; Pempek et al. 2010), suggests that differences in gaze metrics, e.g., durations of fixations, may be task-independent and, therefore, hindering cross-age comparisons of cognitive capacities. Furthermore, before the 18th month, children may have difficulties in understanding video-recorded events (Pempek et al. 2010), which may be an important limitation to developmental studies that test video-recorded stimuli with children younger and older than 18 months and involve cross-age comparisons of performance. Finally, although eye tracking techniques quantify looking behaviors faster and more objectively (Venker and Kover 2015), they are more prone to data loss and may produce different patterns of results, compared to manual gaze coding (Venker et al. 2020). This may be particularly pronounced in developmental studies, as task-irrelevant factors, such as child’s eye color and seating, were shown to correlate with accuracy and data loss in eye tracking (Hessels et al. 2015).

## 6. Summary and Conclusions

In this review paper, three key terms are introduced to organize previous findings from developmental research: simple transfers, complex transfers and flexible problem solving. Both simple and complex transfers involve generalizing knowledge across contexts. However, while simple transfers require inhibiting information that remains irrelevant across these contexts, complex transfers require inhibiting seemingly relevant information in favor of truly relevant information. Although both simple and complex transfers have been repeatedly investigated in children and adolescents, these investigations have not been gathered and bound together before. Since simple and complex transfers draw on executive functions and serve the same function, of rapid and efficient responding to unfamiliar situations, they were gathered under the umbrella term of flexible problem solving.

Flexible problem solving seems to develop in two waves, as the capacity for simple transfers precedes the capacity for complex transfers in development. Generalizing knowledge across mismatching contexts begins to develop early in the first year of life, and rapidly improves between 6 and 30 months. Therefore, between 6 and 30 months, children cope increasingly well with situations that demand inhibiting consistently irrelevant, distracting information. Between 2.5 and 3 years, children’s executive functions responsible for switching improve significantly, but only once this improvement is paired with sufficient goal representation and maintenance around 4 years, children become more ready for complex transfers. Scarce investigations of such transfers suggest that complex transfers may become fully available only at 5 or even 8 years. Flexible problem solving is a key skill in everyday life and the developmental timing of its components, e.g., memory generalization, representational flexibility, task switching, has been extensively investigated in developmental research. However, how children deal with transfers in the presence of conflicting information, whose relevance changes across contexts, remains understudied. This is surprising, given the pedagogical importance of flexible problem solving. Both conceptual knowledge and the skill of prioritizing relevant information over sometimes relevant, but currently relevant information are prerequisites for accurate problem-solving strategies in concrete and abstract problems. Switching between relevant and irrelevant information and recognizing principle for solution across problems is critical to tackling grave challenges, such as climate change (Keen 2010) or societal issues, such as the tension between preference for familiarity vs. openness to unfamiliarity in an increasingly plural and dynamically changing society. Understanding when and how children acquire problem-solving strategies on simpler yet analogical problems should therefore be a priority in education systems that aim to form skilled and responsible members of information societies. Previous research shows that, at early age, children are sensitive to goal-relevant information and, with development, learn to disregard goal-irrelevant information. The integrated approach developed in this paper could guide the development of educational board games, training children in making explicit judgments of goal-relevance and goal-irrelevance of information. Beginner levels in such games could involve solely goal-relevant information, and then, on higher difficulty levels, could, first, introduce goal-irrelevant, distracting information, and, second, goal-irrelevant, misleading information. Alongside such board games, educational booklets and short computer games could be introduced to explain the concept of information relevance in simple, age-appropriate ways, and to encourage incorporating this concept in social, e.g., dramatic, play. Dramatic play, problem solving, citizenship skills and learning to learn are important themes in early childhood education curricula round the world. Since incorporating the concept of goal ir-/relevance of information may support young citizens in making evidence-based decisions, this concept may draw the attention of relevant education stakeholders, from parents and teachers to heads of schools and policy-makers. The integrated approach to flexible problem solving could further inform clinical work, accounting for child’s generalization of visual aids, such as anatomically detailed dolls (e.g., Koocher et al. 1995 in relation to DeLoache and Marzolf 1995). Finally, since previous research points toward an early bilingual advantage in memory generalization and switching, related to speedier improvements in executive functions, age-appropriate trainings of executive functions might, in the future, facilitate earlier development of flexible problem solving.

Focusing on individual problem-solving flexibility has, further, implications for changes in assessment of child’s achievement and progress in the schooling system. Putting emphasis on individual flexibility will, at least to some extent, hinder grouping children into performing below, on and above average and promote focusing on individual course of development instead, both in typically and atypically developing children (Vygotsky 1987). Since flexible problem solving can be investigated with nonverbal methods, it may be tested with clinical populations of children and adults with speech and/or hearing impediments, e.g., in neuropsychological assessment. Furthermore, flexible problem solving, especially with experimental procedures based on looking behaviors, can be investigated in non-human animals to reveal similarities and differences in this capacity between species (for experimental procedures focused on motor behaviors, see Bobrowicz et al. 2020b). Gathering, organizing, and integrating selected findings from several decades of developmental research in this review paper will hopefully facilitate future research on complex transfers, both across immediate and delayed contexts.

## Figures and Tables

**Table 1:** Terms Related to Flexible Problem Solving and Respective Definitions.

Term	Definition	Task Paradigms
Simple transfer	the ability to prioritize relevant over irrelevant, distracting information and apply it to the present situation	A:B::C:D Conceptual problem solving Deferred imitation Object search Physical problem solving Preferential looking Scene analogy Structure mapping task
Complex transfer	the ability to prioritize relevant over irrelevant, misleading information and apply it to the present situation	
Analogical transfer	the ability to detect common principles for solution across problems that share similarity at a deep (e.g., functional) level but differ in the surface format (Crisafi and Brown 1986; p. 954)	A:B::C:D Conceptual problem solving Object search Physical problem solving Preferential looking Scene analogy
Memory flexibility	the ability to generalize information to novel situations (Barr et al. 2020, p. 2); also: a balance between remembering specific features and being able to generalize that knowledge across cues and contexts (Brito and Barr 2014, p. 1157); also: the ability to generalize goal-relevant information retrieved from the existing memory representations across delayed contexts, while disregarding goal-irrelevant information present in the memory representations and in the environment	Deferred imitation Mobile conjugate paradigm
Representational flexibility	inferential use of prior knowledge in new situations; allows retention of goal-relevant information beyond immediate contexts, and its retrieval in a delayed context despite goal-irrelevant information present in both contexts	Deferred imitation Mobile conjugate paradigm
Cognitive flexibility	a broader set of cognitive processes that supports analogical transfer (e.g., Richland and Morrison 2010), and encompasses memory flexibility and representational flexibility along with attentional control, working memory, inhibitory control, and set-shifting/task switching (e.g., Dajani and Uddin 2015); includes set-shifting/task switching, i.e., the ability to flexibly switch between different task dimensions	Dimensional Change Card Sort

**Table 2:** An Overview of Selected Developmental Studies on Simple and Complex Transfers.

Transfer Type	Age	Delay	Verbal Cues	Method	Language	Details	Result	Source
Simple transfer	6 months	30 min	None	Deferred imitation	Monolinguals and bilinguals	Changes within the cue (puppet)	6-month-old monolinguals generalize across a single perceptual change in the cue (color)	Brito and Barr (2014)
							6-month-olds bilinguals generalize across two perceptual changes in the cue (color and shape)	
		24 h	None	Deferred imitation	No information	Changes within the cue (puppet) and the context (location)	6-month-olds fail to generalize across a change in context	Hayne et al. (2000)
							6- month-olds fail to generalize across two perceptual changes in the cue	
						Changes within the cue (puppet), changes within the context (mat ands location)	6-month-olds generalize across a single perceptual change in the context	Learmonth et al. (2004)
							6-month-olds do not generalize to a different-looking cue even if given a chance of immediate imitation	
	9 months	24 h	None	Deferred imitation	No information	Changes within the cue (puppet), changes within the context (mat and slocation)	9-month-olds generalize across two perceptual changes in the context	Learmonth et al. (2004)
							9-month-olds generalize to a different-looking cue, when given a chance of immediate imitation	
						Changes within the cue (button-operated toy), changes within the context (location)	Only crawling 9-month-olds generalize across a simultaneous change in the cue and the context	Herbert et al. (2007)
	10 months	No delay	None	Preferential looking	No information	Generalization of functional knowledge	10-month-olds do not generalize across objects that differ in form but share the same function	Madole et al. (1993)
		60–80 s	Present	Physical problem solving	No information	Combining two source actions to solve a target problem	10-month-olds do not transfer modeled actions unless they receive multiple source problems, or the source and the target are highly perceptually similar	Chen et al. (1997)
	11–12 months	No delay	None	Preferential looking	No information	Generalization of functional knowledge	11–12-month-olds transfer functional knowledge across perceptually dissimilar objects after a demonstration of how one of them works	Träuble and Pauen (2007)
	12 months	10 min	Present	Deferred imitation	No information	Changes within the cue (puppet)	12-month-olds fail to spontaneously generalize across two perceptual changes to the cue (color and shape)	Taylor et al. (2016)
							12-month-olds generalize across two perceptual changes to the cue (color and shape) if they receive a verbal label, either in a familiar or an unfamiliar language, at encoding and retrieval	
							12-month-olds who receive verbal cues at encoding and retrieval show better generalization than 12-month-olds that do not	Herbert (2011)
		24 h	None	Deferred imitation	No information	Changes within the cue (puppet)	12-month-olds fail to generalize across perceptual changes change in color, shape, color and shape	Hayne et al. (1997)
						Changes within the cue (puppet) and the context (location)	12-month-olds generalize across a change in context	Hayne et al. (2000)
							12-month-olds fail to generalize across two perceptual changes in the cue	
	13 months	60–80 s	None	Physical problem solving	No information	Combining two source actions to solve a target problem	13-month-olds transfer modeled actions after fewer and more perceptually dissimilar source problems than 10-month-olds	Chen et al. (1997)
	14 months	No delay	None	Preferential looking	No information	Generalization of functional knowledge	14-month-olds do not generalize across objects that differ in form but share the same function	Madole et al. (1993)
							14-month-olds generalize across objects that differ in form but share the same function	Madole and Cohen (1995)
			Present	Preferential looking	No information	Generalization of functional knowledge	14-month-olds can benefit from verbal cues and transfer functional knowledge across objecsts	Booth and Waxman (2002)
		5 min, 2 days	None	Deferred imitation	No information	Changes within the context (location)	14-month-olds generalize sequences of actions modelled by a peer across changes in the context (location)	Hanna and Meltzoff (1993)
	15 months	10 min	Present	Deferred imitation	No information	Changes within the cue (puppet)	15-month-olds who receive verbal cues at encoding and retrieval show better generalization than 15-month-olds that do not	Herbert (2011)
	16 months	1 week	None	Deferred imitation	No information	Changes within the cue (to functionally equivalent props)	16-month-olds generalize sequences of actions across functionally equivalent props	Bauer and Dow (1994)
	18 months	No delay	None	Preferential looking	No information	Generalization of functional knowledge	18-generalize across objects that differ in form but share the same function	Madole et al. (1993)
				Physical problem solving	No information	Transfer of tool use	18-month-olds fail to generalize functional tool-use knowledge	Pauen and Bechtel-Kuehne (2016)
			Present	Preferential looking	No information	Generalization of functional knowledge	18-month-olds can benefit from verbal cues and transfer functional knowledge across objects	Booth and Waxman (2002)
		10 min	Present	Deferred imitation	No information	Changes within the context (from televised vs. book narrative to physical imitation)	18-month-olds transfer imitation from both meaningful and meaningless televised and book narratives to the same physical props, although better from televised narratives than book narratives, and worse than 24-month-olds	Simcock et al. (2011)
							The narrative, even without visual aids, suffices to facilitate such transfer	
		30 min	None	Deferred imitation	Monolinguals and bilinguals	Changes within the cue (puppet)	Bilingual 18-month-olds are more likely to generalize across two perceptual changes to the cue (color and shape) than monolingual peers	Brito and Barr (2012)
		24 h	None	Deferred imitation	No information	Changes within the cue (puppet)	18-month-olds generalize across two perceptual changes (color and shape) but not if these changes are more significant	Hayne et al. (1997)
						Changes within the cue (puppet) and the context (location)	18-month-olds generalize across a change in context	Hayne et al. (2000)
							18-month-olds generalize well across two perceptual changes in the cue	
			Present	Deferred imitation	Monolinguals and bilinguals	Changes within the cue (to functionally equivalent props)	18-month-old bilinguals but not monolinguals generalize across different-looking but functionally equivalent props, both spontaneously and with a verbal cue at encoding and retrieval	Barr et al. (2020)
					No information	Changes within the cue (to functionally equivalent props)	18-month-olds do not spontaneously generalize a sequence of actions to functionally equivalent, but different-looking props	Herbert and Hayne (2000)
							18-month-olds do not generalize a sequence of actions to functionally equivalent, but different-looking props, even if they receive the same verbal label at encoding and retrieval	
		2 weeks	Present	Deferred Imitation	No information	Changes within the cue (to functionally equivalent props)	18-month-olds generalize a sequence of actions to functionally equivalent, but different-looking props, but narrative at encoding and retrieval did not support performance	Kingo and Krøjgaard (2013)
	20 months	No delay	None	Physical problem solving	No information	Transfer of tool use	20-month-olds fail to generalize functional tool-use knowledge	Pauen and Bechtel-Kuehne (2016)
		1 week	None	Deferred imitation	No information	Changes within the cue (to functionally equivalent props)	20-month-olds generalize sequences of actions across functionally equivalent props	Bauer and Dow (1994)
	21 months	24 h	None	Deferred imitation	No information	Changes within the cue (puppet)	21-month-olds generalize across two, even significant, perceptual changes (color and shape)	Hayne et al. (1997)
	22 months	No delay	None	Physical problem solving	No information	Transfer of tool use	22-month-olds can prioritize functionally relevant over conflicting irrelevant perceptual information but have difficulties in improving performance after feedback	Pauen and Bechtel-Kuehne (2016)
	24 months	No delay	None	Physical problem solving	No information	Transfer of tool use	24-month-olds can prioritize functionally relevant over conflicting irrelevant perceptual information after feedback	Bechtel et al. (2013)
							24-month-olds can prioritize functionally relevant over conflicting irrelevant perceptual information and improve performance after feedback	Pauen and Bechtel-Kuehne (2016)
			Present	Physical problem solving	No information	Combining and transferring two separately learn relationships)	24-month-olds fail to transfer spontaneously across problems	Crisafi and Brown (1986)
		10 min	None	Deferred imitation task	No information	Changes within the context, (from televised vs. book narrative to physical imitation)	24-month-olds transfer imitation from both meaningful and meaningless televised and book narratives to the same physical props, although better from televised narratives than book narratives, and better than 18-month-olds	Simcock et al. (2011)
							The narrative, even without visual aids, suffices to facilitate such transfer	
		24 h	Present	Deferred imitation task	No information	Changes within the cue (to functionally equivalent props)	24-month-olds start to spontaneously generalize a sequence of actions to functionally equivalent, but different-looking props	Herbert and Hayne (2000)
							24-month-olds generalize a sequence of actions to functionally equivalent, but different-looking props if they receive the same verbal label at encoding and retrieval	
	24–30 months	5 min 24 h	None	Physical problem solving	No information	Transfer of tool use	24- to 30 months do not transfer tool use from a source to a target	Bobrowicz et al. (2020a)
	30 months (2.5 years)	5 min 24 h	None	Physical problem solving	No information	Transfer of tool use	2.5-year-olds are equally likely to transfer tool use across 5 min and 24 h	Bobrowicz et al. (2020a, 2022)
		No delay 24 h 1 week	Present	Object search	No information	Transfer across models and physical spaces	2.5-year-olds transfer relationships between objects in the presence of perceptual dissimilarities, even after 1 week	DeLoache et al. (2004)
		24 h	Present	Deferred imitation	No information	Changes within the cue (to functionally equivalent props)	30-month-olds spontaneously generalize a sequence of actions to functionally equivalent, but different-looking props	Herbert and Hayne (2000)
	2.5–3.5 years	None	Present	Object search	No information	Transfer across models and physical spaces	Between 2.5 and 3.5, children require a lower and lower degree of similarity across objects and scene size to transfer object relationships across perceptually dissimilar scenes	DeLoache et al. (1991)
	3 years	None	Present	Object search	No information	Transfer across models and physical spaces	3-year-olds can transfer relationships between objects of low perceptual similarity if given minimal instructions	DeLoache et al. (1999)
				Physical problem solving	No information	Combining and transferring two separately learn relationships)	3-year-olds can transfer a solution across physically similar problems	Crisafi and Brown (1986)
							3-year-olds can combine and transfer separately learned relationships	Halliday (1977)
				Conceptual problem solving	No information	Transfer across stories on novel use of familiar tools and biological concepts	3-year-olds transfer knowledge across age-appropriate stories	Brown et al. (1989)
							3-year-olds can transfer knowledge between source and target but require more experience with transfers than 4-year-olds	Brown and Kane (1988)
							3-year-olds are more likely to generalize across problems when prompted to discuss similarity than otherwise	
							3-year-olds spontaneously mention similarities across problems when teaching a puppet, and thereafter are more likely to generalize across these problems than if they are externally prompted to discuss similarity or told that such similarity across problems exists	
				A:B::C:D	No information	Transfer across pictures	3-year-olds can transfer knowledge about relationships across causally clear problems	Goswami and Brown (1989)
	4 years	None	Present	Physical problem solving,	No information	Combining and transferring two separately learn relationships	4-year-olds can transfer across physically dissimilar problems	Crisafi and Brown (1986)
				Conceptual problem solving	No information	Transfer across stories on novel use of familiar tools and biological concepts	4-year-olds generalize across problems spontaneously, without being prompted	Brown and Kane (1988)
				Object search	No information	Transfer across models and physical spaces	4-year-olds can transfer relationships between objects of higher perceptual dissimilarity and with fewer instructions than 3-year-olds	DeLoache et al. (1999)
				A:B::C:D	No information	Transfer across pictures	4-year-olds can transfer knowledge about relationships across causally clear problems	Goswami and Brown (1989)
						Transfer across pictures	4-year-olds can detect and transfer relationships between objects based on shape sand proportion	Goswami (1989)
	4–5 years	No delay	Present	Structure mapping task	No information	Transfer across metaphors	4–5-year-olds can equally well map relations between objects onto functionally equivalent objects as 7-year-olds	Gentner (1977)
		7 months	Present	A:B::C:D	No information	Transfer across pictures	4–5-year-olds can detect and transfer relationships between objects based on color, shapes and size	Alexander et al. (1989)
	4–6 years	No delay	Present	Physical problem solving	No information	Planning divergent strategies of solving a physical target problem based on a source story	4-to-6-year-olds can generalize simple relationships from a source story to a target physical problem, even when these problems are highly perceptually dissimilar	Holyoak et al. (1984)
							4- and 6-year-olds have difficulties in transferring incomplete relationships between a source and a target	
	5 years	No delay	None	A:B::C:D	No information	Transfer across pictures	5-year-olds focus more on the C than on the relation between A and B compared to 13-year-olds	Thibaut and French (2016)
	5–6 years	No delay	Present	Physical problem solving	No information	Combining and transferring two separately learn relationships	5–6-year-olds can combine and transfer separately learned relationships	Hewson (1978)
				Physical and conceptual transfers	No information	Solving physical targets after source stories with superficial, structural, procedural similarities	5- and 6-year-olds transfer knowledge across problems that differ in terms of superficial, structural and procedural features	Chen (1996)
	5–7-years	No delay	Present	Object search	No information	Transfer across models and physical spaces	5–7-year-olds can transfer relationships between objects in the presence of high perceptual dissimilarity and without any instruction	DeLoache et al. (1999)
	6 years	No delay	Present	A:B::C:D	No information	Transfer across pictures	6-year-olds can transfer knowledge about relationships across causally clear problems	Goswami (1989)
	6–7 years	No delay	Present	Physical and conceptual transfers	No information	Solving a physical target after source stories with the same or different solution principle	6–7-year-olds spontaneously transfer analogous solutions as long as base and target problems share some surface similarities; negative transfer occurs when the solution principle differs between base and target problems	Chen and Daehler (1989)
	8 years	No delay	None	A:B::C:D	No information	Transfer across pictures	8-year-olds focus more on the on the C than on the relation between A and B compared to 13-year-olds	Thibaut and French (2016)
	8 years	No information	Present	A:B::C:D	Inconclusive, toward bilingual (children learnt both English and Hebrew)	Transfer across pictures	8-year-olds fail to detect a higher-order relationship between two separate but functionally equivalent relationships	Sternberg and Rifkin (1979)
	10 years	No information	Present	A:B::C:D	Inconclusive, rather bilingual (children learnt both English and Hebrew)	Transfer across pictures	10-year-olds detect a higher-order relationship between two separate but functionally equivalent relationships	Sternberg and Rifkin (1979)
	12 years	No information	Present	A:B::C:D	Inconclusive, toward bilingual (children learnt both English and Hebrew)		12-year-olds detect a higher-order relationship between two separate but functionally equivalent relationships	Sternberg and Rifkin (1979)
Complex transfer	3 years	No delay	Present	Scene analogy	No information	Relational complexity vs. featural distraction in picture sets	3-year-olds transfer knowledge about one relationship in the absence of misleading information	Richland et al. (2006)
							3-year-olds have difficulties in transferring knowledge across two relationships, even in the absence of misleading information and in the presence of explicit verbalization of the relationships	
	4 years	No delay	Present	Scene analogy	No information	Relational complexity vs. featural distraction in picture sets	4-year-olds transfer knowledge about one relationship in the absence of misleading information	Richland et al. (2006)
							4-year-olds have difficulties in transferring knowledge across two relationships, even in the absence of misleading information and in the presence of explicit verbalization of the relationships	
	5–6 years	No delay	Present	A:B::C:D	No information	Goal irrelevance vs. salience in picture sets	5- to 6-year-olds benefit from verbalizing a relation between two items when finding the same relational match for another item as long as the relation is goal-relevant; Performance is impaired if a goal-irrelevant yet salient relation between two items is verbalized before finding the same relational match for another item	Glady et al. (2017)
	6 years	No delay	None	A:B::C:D	No information	Relational complexity vs. featural distraction in picture sets	6-year-olds misunderstand the task	Thibaut et al. (2010)
	6–14 years	No delay	Present	Scene analogy	No information	Relational complexity vs. featural distraction in picture sets	With age, 6-to-14-year-olds were less and less affected by relational complexity and misleading information	Richland et al. (2006)
	8 years	No delay	None	A:B::C:D	No information	Relational complexity vs. featural distraction in picture sets	8-year-olds generalize functional relationships but are not immune to perceptual distraction	Thibaut et al. (2010)
	14 years	No delay	None	A:B::C:D	No information	Relational complexity vs. featural distraction in picture sets	14-year-olds generalize functional relationships and immune to perceptual distraction	Thibaut et al. (2010)

## Data Availability

All data is available in the manuscript.

## Note

1 Note that the construct of cognitive flexibility cannot be reduced to either the context of executive functions, or to set-shifting/task-switching. This term has been defined differently across studies focusing on behavior, or memory, or attention. In terms of behavior, cognitive flexibility refers to the capacity to modify or adjust one’s behavior in response to changes in the environment or task requirements, for instance, to switch between different behavioral responses or strategies to effectively navigate and adapt to new situations (e.g., Morand-Ferron et al. 2022; Tello-Ramos et al. 2019; also termed behavioral flexibility, e.g., Uddin 2021). In terms of memory, cognitive flexibility refers to the ability to update, reorganize, or modify existing memory representations in order to integrate new knowledge with previously learned information and adjust memory retrieval processes accordingly (e.g., Koslov et al. 2019). Finally, in terms of attention, cognitive flexibility is conceptualized as a core executive function, which supports detection of changing rules in the environment and adjustment of one’s own behavioral responses (e.g., Bunge and Zelazo 2006). In this context, set-shifting or task switching, involving higher-order stimulus-response rules for selecting currently relevant task sets, are the most complex form of cognitive flexibility (e.g., Dajani and Uddin 2015; Uddin 2021). Cognitive flexibility in this context aids also handling simpler rules, such as reversing stimulus-reward associations and pairs of conditional stimulus-response rules (e.g., Bunge and Zelazo 2006).
